# A Case of Chronic Cirrhotic Kidney

**Published:** 1886-10

**Authors:** H. McHatton

**Affiliations:** Macon, Ga.


### Clinic Reports.

### A CASE OF CHRONIC CIRRHOTIC KIDNEY.

REMARKS BEFORE THE MACON MEDICAL SOCIETY, SEPTEMBER 2D, 1886.

By H. McHATTON, M. D., Macon, Ga.

Reported for The Atlanta Medical and Surgical Journal.

Last night, at 9 o'clock, I was called to see Mr. C., American, aged 40, of good family and personal history. I found him suffering from nausea—he had been vomiting more or less all day. Pulse 70, hard and full, temperature normal. Left ventricle slightly enlarged, muscular element of first sound decidedly accentuated. Some dizziness. Said he often had specks before his eyes, and that after being on his feet all day they were slightly swollen. Kidneys acting well. Had noticed nothing unusual about his urine. These attacks came on about once a month, and he has a feeling of general malaise between times. He has seen three physicians; two of them examined his urine and said there was nothing the matter with it. All gave him our favorite diagnosis, which is even more fashionable than our Northern brother's malaria, namely, biliousness. I gave him a mixture of bismuth and spt. ammon. aromat. to quiet his stomach, and took a specimen of urine for analysis, which gave the following results: Color, pale and clear; sediment, very slight; odor, normal; reaction, neutral; specific gravity, 1012; heat and nitric acid test, negative; Heller's test gave a very faint, cloudy line; hyaline and granular casts, both large and small, abundant.

From this examination and the history, I made a diagnosis of chronic cirrhotic kidney. On my visit, at 11 a. m. to-day, I found him in uræmic coma, from which he could be aroused with difficulty, and when aroused was incoherent. He had fallen out of bed during the morning, injuring his face and head, besides biting his tongue badly, of which fact he was not conscious. I gave him the following prescription:

℞. Hyd. chlo. mit.

Soda bicarb .......................aa grs. x

Chart No. 1.

S. One dose.

℞. Kali acet............................℥ ss.
Inf. digital...............................℥ iii.
M. Sig. Teaspoonful every two hours.

On my visit at seven to-night I found him in perfect possession of his faculties, bowels and kidneys acting freely; in fact, he is as well as usual, and barring intercurrent affections will continue so until he gets another accumulation of urea.

This case is a true type of the most insidious and probably the most frequent of all the kidney diseases that we group for convenience under the name of chronic Bright's. It is far more common than we suppose, this being the third of the kind that I have seen lately. It is in this class of cases that we get the sudden and often fatal manifestations of uræmia with little or no premonition. They do not give the train of symptoms that we are in the habit of associating with Bright's.

The urine in gross appearance is usually normal; the quantity is often in excess, but the specific gravity is usually low. Albumen is rarely abundant and often entirely absent. Dropsies are as a rule absent, and if present are not of much importance. In fact, there is rarely any symptom that will invite our attention to the kidneys if we are not especially on our guard.

Our patient usually presents himself complaining of a general sense of malaise, or with one of the complications of his disease. A general sense of failing health with hypertrophy of the left ventricle without valvular lesions and urine of low specific gravity in a patient of middle age should always induce us to make careful and repeated examinations of the urine.

In regard to the treatment of these cases, the damage to the organ is done and we cannot reproduce the destroyed part, but even if the secreting substance is destroyed to the extent of the bulk of one kidney, the condition is not incompatible with life, or even a fair state of health; by judicious management we can often guide these cases through years of usefulness.

There is of course no special medication for the disease proper, and our dependence is on hygiene and tonics, of which, in view of the existing state of anæmia, iron should be the principal. If our patient can be induced to use it, it is best to furnish him with a urinometer, and get him to take the specific gravity and quantity of his urine every few days. By taking these two factors (neither one being of absolutely no value alone), a glance at the table in most of the works on urinary analysis will show us if he is passing sufficient urea; as long as he is doing so let your diuretics severely alone, as they are at that time not only useless, but even contra-indicated, for by stimulating the kidneys when there is no call for it, we will be less liable to get a response to our medication when it is of vital importance. The same line of treatment holds good in respect to the heart; so long as the hypertrophy has not reached its limit, it is bad judgment to use cardiac stimulants (excepting in cases of urgent necessity during an accumulation of urea), for eventually, should the patient live, this condition of hypertrophy will be followed by dilatation, and then we will have urgent need of our cardiac stimulants.

